# Translating research to usual care of children with sickle cell disease in Northern Nigeria: lessons learned from the SPRING Trial Team

**DOI:** 10.1186/s13104-021-05885-1

**Published:** 2022-01-04

**Authors:** Halima Bello-Manga, Lawal Haliru, Abdulkadir M. Tabari, Bilkisu Farouk, Aisha Suleiman, Gloria Y. Bahago, Abdulrasheed M. Sani, Ana A. Bauman, Michael R. DeBaun, Allison A. King

**Affiliations:** 1 Department of Hematology and Blood Transfusion, Barau Dikko Teaching Hospital/Kaduna State University, Lafia Road, Kaduna, Nigeria; 2 Department of Pediatrics, Barau Dikko Teaching Hospital/Kaduna State University, Kaduna, Nigeria; 3 Department of Radiology, Barau Dikko Teaching Hospital/Kaduna State University, Kaduna, Nigeria; 4 Department of Nursing Services, Hematology Unit, Barau Dikko Teaching Hospital/Kaduna State University, Kaduna, Nigeria; 5 Research Office, Barau Dikko Teaching Hospital/Kaduna State University, Kaduna, Nigeria; 6 Department of Surgery, Washington University in St. Louis, St. Louis, Missouri USA; 7 Department of Pediatrics, Division of Pediatric Neurology, Vanderbilt University of Medicine, Nashville, Tennessee USA; 8 Department of Pediatrics, Division of Hematology and Oncology, Washington University in St. Louis School of Medicine, St. Louis, Missouri USA

**Keywords:** Stroke, Sickle cell disease, PRISM

## Abstract

**Objectives:**

Evidence-based practice for stroke prevention in high-income countries involves screening for abnormal transcranial Doppler (TCD) velocity and initiating regular blood transfusions for at least 1 year, followed by treatment with hydroxyurea. This practice has not been transferred to low-resource settings like Nigeria, the country with the highest global population density of SCD. Following a multi-center randomized controlled trial among children with SCA in northern Nigeria, screening for stroke and initiation of hydroxyurea was established as standard of care at the clinical trial sites and other locations. We aim to describe the critical steps we took in translating research into practice for stroke prevention in SCA in Nigeria. Guided by the PRISM framework, we describe how we translated results from a randomized controlled trial for primary prevention of stroke in children with sickle cell anemia into usual care for children with SCA in Kaduna, Nigeria.

**Results:**

Findings from this study demonstrate the importance of organizational support and stakeholder involvement from the onset of a clinical trial. Having the dual objective of conducting an efficacy trial while simultaneously focusing on strategies for future implementation can significantly decrease the lag time between discovery and routine practice.

## Introduction

Sickle cell anemia (SCA), an inherited blood disorder, is the leading cause of stroke in children. An estimated 11% of unscreened and untreated children with SCA are at increased risk of stroke and will have at least one stroke by 17 years of age [1]. The evidence-based practice (EBP) for primary stroke prevention in children with SCA involves screening for abnormal transcranial Doppler ultrasound (TCD) velocity (> 200 cm/s). Patients with abnormal TCD velocity of 200 cm/s or higher receive blood transfusion therapy for at least 1 year followed by treatment with hydroxyurea [2]. This EBP decreases the risk of stroke by 92% [3], leading to a tenfold drop in stroke incidence. This well-established EBP for stroke prevention in children with SCA is not implemented in low middle-income countries (LMICs), such as Nigeria, where more than 50% of the world’s 300,000 children with SCA are born [4].

## Main text

### Methods

This paper presents a case study, highlighting the critical steps taken in translating research into practice for stroke prevention in SCA in Nigeria. Few publications share experiences in planning for implementation while conducting effectiveness sickle cell research in LMICs, and we argue that sharing these details is critical for investigators in similar countries to learn from these efforts. When implementing EBP in low resource settings, much happens in terms of establishing the infrastructure so a trial can be successful and not a “Trojan Horse” for the community [5]. In other words, when implementing an EBP in different contexts, it is crucial to the thoughtful of limited resources. Engaging in international efforts to disseminate and implement EBPs can be important to improve healthcare and health disparities, but unless it is done with close and equitable collaboration with local stakeholders, implementing EBPs may contribute to deviations that result in the poor application of both intervention and research methods to the detriment of participants and science. Below, we share how the implementation of two trials resulted in changing the healthcare system for children with SCA in Kaduna, northern Nigeria.

#### Conceptual framework

**Fig. 1:**
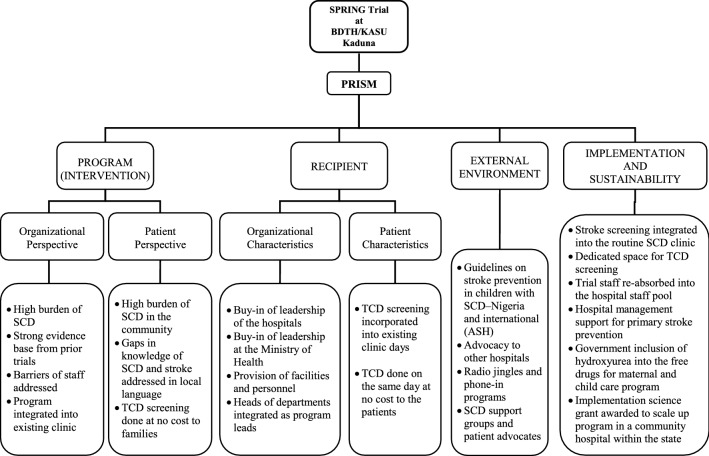
PRISM elements activated in translating research to practice following the SPRING trial in an academic hospital in Kaduna, Nigeria

Understanding the contextual factors is necessary to integrate EBP into usual practice [6]. Our work is guided by the Practical Robust Implementation and Sustainability (PRISM) Model, which has four domains; (1) the program (intervention), (2) the recipient, (3) the external environment, and (4) implementation and sustainability infrastructure. Figure 1 shows how we used PRISM following the SPRING (NCT02560935) trial. Below, we describe our work.

##### *Program (Intervention)*

Despite the high stroke burden within the region, there were no stroke prevention strategies for children with SCA in Kaduna. Barriers to stroke prevention strategies included a lack of TCD services [7], primarily due to lack of trained personnel certified in performing TCD, shortages of machines, and lack of safe blood for transfusion [8]. We also needed to examine whether hydroxyurea therapy was an acceptable EBP for primary stroke prevention in children with an abnormal TCD. To answer this question, in 2011, our feasibility trial demonstrated high participant recruitment, retention, and adherence rates for hydroxyurea for primary stroke prevention in children with SCA [8]. Further, we completed a multi-center trial (SPRING, NCT02560935) in three centers in two states, Kano and Kaduna, to examine the optimal dose of hydroxyurea (20 mg/kg vs. 10 mg/kg) for primary stroke prevention. Based on these results, the American Society of Hematology (ASH) guidelines recommended that children with SCA and abnormal TCD living in LMICs receive at least moderate-dose (20 mg/kg per day) hydroxyurea [9].

##### *The recipient*

Before our randomized controlled trial, families had little knowledge regarding SCA and the associated complications. There was little organizational infrastructure for stroke prevention in local health facilities, including the academic hospital. To run the trial, we secured buy-in from hospital leadership and assembled a team. While the site awaited approval from the national and local institutional review boards, the team was certified in research governance, including ethical conduct of research, good clinical practice, and NIH training in research on human participants and how to conduct audits.

At the **organizational level**, securing the commitment of top management of the hospital for the trial was essential. The hospital leadership appropriated clinic space, personnel, and other hospital facilities for the study. All team members, including the research coordinators, research assistants, phlebotomists, pharmacists, nurses, medical officers, and medical monitors, participated in regular weekly meetings with the Principal Investigator and other members of the wider research team to address any problems encountered during the week. The stroke prevention program was incorporated into the existing SCA clinic, which ran weekly.

To address the lack of TCD screening services, the research leadership provided TCD machines and organized trainings on the conduct of TCD and stroke detection. Given the shortage of radiologists in the hospital, we trained non-specialist medical officer alongside a radiologist to conduct TCD screenings, a more sustainable option than training a radiologist alone. Conducting TCD screening on clinic days and those with abnormal TCD to be evaluated by the pediatrician on the same day helped reduce the burden of having to return at a later date for parents and patients, including the cost of transportation, hours spent out of work for parents, and missed school days for the children.

Having recognized the knowledge gap regarding SCA and stroke among families of children with SCA, we considered the **patient characteristics** to develop general education materials on SCA, emphasizing stroke signs and symptoms, including videos and pamphlets in Hausa language, the region’s indigenous language. With the support of the hospital leadership and the State’s Ministry of Health, radio jingles were aired on local radio stations describing stroke as a complication of SCA, how it can be prevented, and the availability of TCD screening at the academic hospital. Additionally, patients were able to call the clinic directly to book appointments for TCD screenings.

##### *External environment*

Working closely with Kaduna’s state leadership was imperative for implementing the SCA guidelines. Their endorsement of the stroke screening programs was essential to disseminate information and raise awareness about the screenings. Other health care facilities within the state were informed of these guidelines and the stroke prevention program at the academic hospital through the leadership of the hospitals, and radio announcements was used to create awareness to the public.

##### *Implementation and sustainability infrastructure*

After the completion of our SPRING trial, TCD screening became standard of care, and children with abnormal TCD values were identified and prescribed hydroxyurea. Unfortunately, the team realized that most of the families could not afford hydroxyurea; therefore, we developed stop-gap strategies to provide hydroxyurea for these children, which included; (a) raising money from within the teams’ personal resources and from local philanthropists and (b) the leadership of the academic hospital taking responsibility for providing free hydroxyurea and some laboratory investigations for the children with abnormal TCD values for 1 year.

We met with officials at the Health Ministry to deliberate on the best strategy for ensuring the availability and sustainability of hydroxyurea for the children after completion of enrollment for the trial. After significant advocacy, the governor of Kaduna State approved the provision of free hydroxyurea for children with abnormal TCD values for 5 years. More recently, the leadership of the agency responsible for the procurement, warehousing, and distribution of health commodities, has recognized the role of hydroxyurea in the prevention of stroke among children with SCA. As such, the agency has included the provision of hydroxyurea into the state’s free maternal and child drug package, thereby making it accessible at no cost to families. To ensure transparency and accountability of the government’s free hydroxyurea, we established an e-prescription system, which provides a platform for periodic audits.

Given that our initial study was conducted in an academic hospital, we have secured funding from the NIH to initiate a stroke prevention program in a community hospital by task shifting of TCD screening to nurses. Currently, we plan to scale up the stroke prevention program to other parts of the state.

#### Impact of the SPRING trial on the care of children with SCA in Kaduna, Nigeria

Before the SPRING trial, *no TCD was done in Kaduna*. Capacity building was incorporated at the beginning of the trial, therefore, immediately after enrolment was completed, for the trial, TCD screening became standard of care and children with abnormal TCD values were identified and prescribed hydroxyurea. From 2017 to October, 2021, a total of 2005 TCD examinations were conducted, out of which 123 were found to be abnormal (> 200 cm/s) and 98 of these children have been receiving hydroxyurea free of charge (Table 1). Currently, by estimation, the ‘***reach***’ for TCD in Kaduna metropolis (defined as the number of children that had TCD measurement divided by the total number of eligible children) is 10% (2005/20,040), which is quite low. Given that our initial study was conducted in an academic hospital, we were funded by Fogarty International Center of the NIH (K43TW011583) to replicate a similar program as the one at the academic center at a community hospital in a densely populated part of Kaduna town where majority of the patients with SCD seek care. To sustain this effort, we plan on scaling up the stroke prevention program to involve other parts of Kaduna State and eventually, the whole of Nigeria.

**Table 1:** Summary of TCD examinations conducted at Barau Dikko Teaching Hospital over 5 years (2017–2021)

Year	2016	2017	2018	2019	2020	2021	Total
Total number of TCDs performed	0	456	550	375	216	408	2005
Total number of abnormal TCDs	0	21	29	23	18	32	123

### Discussion

We have described how the PRISM model was used to identify contextual factors in the planning, implementation, and sustainability of our research-oriented program on stroke prevention in children with SCD in Kaduna, Nigeria, and its integration into the usual care of this patient population at an academic hospital (Barau Dikko Teaching Hospital/Kaduna State University), one of the sites for the SPRING trials. Research findings are generated in controlled settings and often fail to be fully utilized in real-world settings because contextual factors that enhance the intervention’s uptake are not appropriately addressed [10]. Previous studies have shown the importance of considering multi-level context and contextual factors a priori and throughout the dissemination and implementation process, as the context is always changing [11–14]. To decrease the difference in the lag time between completion of a phase III randomized controlled trial and adoption in the community, which is typically 17 years [15], we have demonstrated the importance of implanting a sustainability strategy prior to the start of a randomized controlled trial in this paper.

### Limitations

**Fig. 2:**
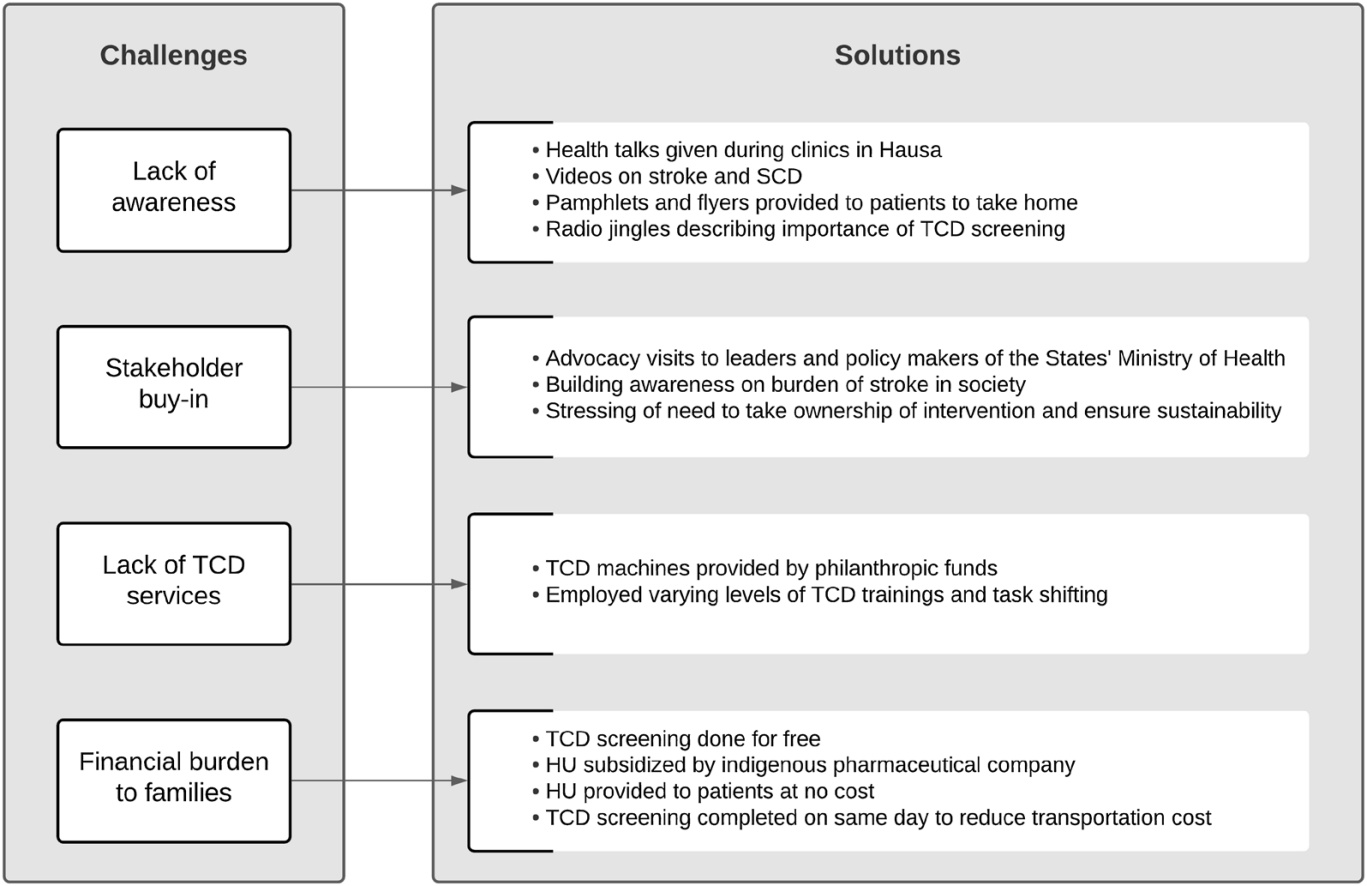
Challenges to establishing a stroke prevention program in northern Nigeria and how they were addressed. (*HU* hydroxyurea, *TCD* Transcranial Doppler)

One of the significant challenges we faced in these trials included frequent changes in the leadership of the Ministry of Health. Some of the senior policymakers had no knowledge of stroke in children, particularly those with SCA, and were skeptical about the intervention. To address these challenges, we engaged with leadership and policymakers whenever there was a change in the administrative direction by conducting advocacy visits and briefs. Additionally, we partnered with a ‘champion’ of our work at the Ministry of Health, who would provide updates and follow up on previous discussions. A summary of the challenges we faced and how they were addressed is presented in Fig. 2. Currently, this effort is being scaled up to go beyond our teaching hospital to other community hospitals within the state.

Applying implementation science methods to improve the uptake EBPs in LMICs allows researchers to create resources, change behavior, use creative and innovative methods, build research capacity, and push for policy changes to support the science. With an increase in funding for SCA-related research, it is our hope that, by utilizing the tools of implementation science at the onset of the randomized controlled trials with ongoing weekly assessment and critical evaluations, we can promote uptake of evidence-based care for children with SCA.

## Data Availability

The datasets used during the current study are available from the corresponding author on reasonable request.

## Abbreviations

TCD: Transcranial Doppler velocity
SCD: Sickle cell disease
SCA: Sickle cell anemia
PRISM: Practical Robust Implementation and Sustainability Model
EBP: Evidence-based practice
LMICs: Low middle income countries
SPRING: Primary Prevention of Stroke in Children with Sickle Cell Anemia in Sub-Saharan Africa
ASH: American Society of Hematology
NIH: National Institutes of Health
BDTH/KASU: Barau Dikko Teaching Hospital/Kaduna State University
HU: Hydroxyurea
